# Supplementary material for: Hexameric helicase G40P unwinds DNA in single base pair steps
Source: eLife. 2019 Jan 28;8:e42001. doi: 10.7554/eLife.42001 (PMC6370340; doi:10.7554/eLife.42001)
Supplement: Supplementary file 1. [file elife-42001-supp1.docx]

Supplementary File 1

**Supplementary Tables**

| **Substrate** | **40 AT** | **40 AT** | **39 AT 1 GC** | **39 AT 1 GC** | **38 AT 2 GC** | **38 AT 2 GC** | **37 AT 3 GC** | **37 AT 3 GC** |
| --- | --- | --- | --- | --- | --- | --- | --- | --- |
| **[G40P_6_]=60nM** | + | + | + | + | + | + | + | + |
| **[DnaG]=180nM** | - | + | - | + | - | + | - | + |
| **Full unwinding [%]** | 89 | 84 | 69 | 77 | 57 | 73 | 12 | 58 |
| **# of unwinding** | 112 | 88 | 69 | 70 | 60 | 53 | 13 | 52 |
| **# of attempts** | 14 | 17 | 30 | 21 | 46 | 20 | 94 | 38 |

**Table S1:** **Unwinding of G40P at room temperature (21ºC) in presence and absence of primase DnaG at [ATP] = 1 mM**

| **Substrate** | **40 AT** | **40 AT** | **39 AT 1 GC** | **39 AT 1 GC** | **38 AT 2 GC** | **38 AT 2 GC** | **37 AT 3 GC** | **37 AT 3 GC** |
| --- | --- | --- | --- | --- | --- | --- | --- | --- |
| **[G40P_6_]=60nM** | + | + | + | + | + | + | + | + |
| **21ºC** | + | - | + | - | + | - | + | - |
| **33ºC** | - | + | - | + | - | + | - | + |
| **Full unwinding [%]** | 89 | 87 | 70 | 77 | 56 | 72 | 12 | 63 |
| **# of unwinding** | 112 | 166 | 69 | 27 | 60 | 74 | 13 | 66 |
| **# of attempts** | 14 | 24 | 30 | 8 | 46 | 28 | 94 | 39 |

**Table S2: Unwinding G40P at different experiment temperatures at [ATP] = 1 mM**

| **[ATP] in µM** | **[ADP] in µM** | **DnaG [180 nM]** | **Avg slips** |
| --- | --- | --- | --- |
| 1000 | 0 | - | 0.66 |
|  | 0 | + | 0.26 |
|  | 500 | - | 0.68 |
|  | 1000 | - | 0.54 |
| 500 | 0 | - | n.d. |
|  | 0 | + | 0.31 |
|  | 500 | - | 1.14 |
|  | 1000 | - | 1.02 |
| 250 | 0 | - | 1.17 |
|  | 0 | + | 0.39 |
|  | 500 | - | 1.58 |
|  | 1000 | - | 1.58 |
| 150 | 0 | - | 1.73 |
|  | 0 | + | 0.53 |
|  | 500 | - | n.d. |
|  | 1000 | - | n.d. |
| 100 | 0 | - | 2.05 |
|  | 0 | + | n.d. |
|  | 500 | - | 1.64 |
|  | 1000 | - | n.d. |
| 75 | 0 | - | 2.42 |
|  | 0 | + | 0.85 |
|  | 500 | - | n.d. |
|  | 1000 | - | n.d. |

**Table S3: Average number of slips before successful unwinding**

|  | **unwinding** | **no unwinding** |
| --- | --- | --- |
| **no anti-Dig** | 115 | 159 |
| **anti-Dig** | 11 | 221 |

**Table S4: Number of traces with or without unwinding in the anti-Dig experiment**

| **Name** | **Sequence (5’ to 3’)** |
| --- | --- |
| AT-Cy3 | (T30)\iAmMC6T\AAT TAT ATT TAA ATT TAA ATA TTA ATT AAT ATA TTA ATA T |
| Dig-AT-Cy3 | \5DigN\TTT TTT TTT TTT TTT TTT TGG CTT CGG TGG\iAmMC6T\AAT TAT ATT TAA ATT TAA ATA TTA ATT AAT ATA TTA ATA T |
| AT-Cy5 | \5BiosG\ATA TTA ATA TAT TAA TTA ATA TTT AAA TTT AAA TAT AAT \iAmMC6T\(T30) |
| AT1GC-Cy5 | \5BiosG\AT ATT AAT ATA TTA ATT AAT ATT TAA ATT CAA ATA TAA T\iAmMC6T\(T30) |
| AT1GC-Cy3 | (T30)\iAmMC6T\ AA TTA TAT TTG AAT TTA AAT ATT AAT TAA TAT ATT AAT AT |
| AT2GC-Cy5 | \5BiosG\AT ATT AAT ATA TTA ATT AAT ATT TAA ATG CAA ATA TAA T\iAmMC6T\(T30) |
| AT2GC-Cy3 | (T30)\iAmMC6T\AA TTA TAT TTG CAT TTA AAT ATT AAT TAA TAT ATT AAT AT |
| AT3GC-Cy5 | \5BiosG\AT ATT AAT ATA TTA ATT AAT ATT TAA AGG CAA ATA TAA TT\iAmMC6T\(T30) |
| AT3GC-Cy3 | (T30)\iAmMC6T\AA TTA TAT TTG CCT TTA AAT ATT AAT TAA TAT ATT AAT AT |
| 4AT4GC-Cy5 | \5BiosG\CGC GAT TAC GGC AAT TCC GGA ATA CCG CTA ATG GCC TTA \iAmMC6T\(T30) |
| 4AT4GC-Cy3 | (T30)\iAmMC6T\AT AAG GCC ATT AGC GGT ATT CCG GAA TTG CCG TAA TCG CG |
| 7AT33GC-Cy5 | \5BiosG\CGC CCG CGC CCG CCG CCC CGC CCG CCC CGC GGC TAA TAA \iAmMC6T\(T30) |
| 7AT33GC-Cy3 | (T30)\iAmMC6T\AT TAT TAG CCG CGG GGC GGG CGG GGC GGC GGG CGC GGG CG |
| 10AT30GC-Cy5 | \5BiosG\CGC CCG CGC CCG CCG CCC CGC CCG CCC CGC AAA TAA TAA \iAmMC6T\(T30) |
| 10AT30GC-Cy3 | (T30)\iAmMC6T\AT TAT TAT TTG CGG GGC GGG CGG GGC GGC GGG CGC GGG CG |
| 13AT27GC-Cy5 | \5BiosG\CGC CCG CGC CCG CCG CCC CGC CCG CCC TAT AAA TAA TAA \iAmMC6T\(T30) |
| 13AT27GC-Cy3 | (T30)\iAmMC6T\AT TAT TAT TTA TAG GGC GGG CGG GGC GGC GGG CGC GGG CG |
| AT-tracking | (T31)AAT TAT ATT TAA ATT TAA ATA TTA ATT AAT ATA TTA ATA T\3Bio\ |
| AT-non-tracking | ATA TTA ATA TAT TAA TTA A\iAmMC6T\A TTT AAA TTT AAA TAT AAT \iCy5\(T30) |

**Table S5: DNA oligo sequences**
